# A New Local Modelling Approach Based on Predicted Errors for Near-Infrared Spectral Analysis

**DOI:** 10.1155/2016/5416506

**Published:** 2016-06-30

**Authors:** Haitao Chang, Lianqing Zhu, Xiaoping Lou, Xiaochen Meng, Yangkuan Guo, Zhongyu Wang

**Affiliations:** ^1^School of Instrumentation Science & Opto-Electronics Engineering, Beihang University, Beijing 100191, China; ^2^Beijing Key Laboratory for Optoelectronic Measurement Technology, Beijing Information Science & Technology University, Beijing 100192, China

## Abstract

Over the last decade, near-infrared spectroscopy, together with the use of chemometrics models, has been widely employed as an analytical tool in several industries. However, most chemical processes or analytes are multivariate and nonlinear in nature. To solve this problem, local errors regression method is presented in order to build an accurate calibration model in this paper, where a calibration subset is selected by a new similarity criterion which takes the full information of spectra, chemical property, and predicted errors. After the selection of calibration subset, the partial least squares regression is applied to build calibration model. The performance of the proposed method is demonstrated through a near-infrared spectroscopy dataset of pharmaceutical tablets. Compared with other local strategies with different similarity criterions, it has been shown that the proposed local errors regression can result in a significant improvement in terms of both prediction ability and calculation speed.

## 1. Introduction

Near-infrared (NIR) spectroscopy plays an important role in the analysis of complex samples or chemical process due to its simplicity, rapidity, and nondestructive measurements [1–4]. As a result, multivariate calibration methods that relate property (*Y*) and spectra (*X*) have been extensively used in the quantitative analysis of NIR spectroscopy. Many authors have stated that the choice of the appropriate calibration method is one of the key factors that influence the performance in prediction of the property *Y* of query samples [5].

The multivariate calibration methods, such as multiple linear regression (MLR), principal component regression (PCR) [6], and partial least squares (PLS) [7] regression, have been adopted for the first time [2, 8]. However, these methods are based on statistical linear models which are not always met in real-life situations and therefore are not able to efficiently model the nonlinear relationship between *Y* and *X*. To solve this problem, different authors have demonstrated that nonlinear algorithms such as Artificial Neural Network (ANN) [9] and Least Squares Support Vector Machines (LS-SVM) [10] can produce better results than traditional linear methods especially used together with large NIR spectral libraries [11].

The approach presented in this paper is local method, which has been widely used in near-infrared spectroscopy analysis because of its advantages in terms of the simplicity of the model constructed and the ability to cope with nonlinearities [12–14]. The essential idea of local method is to develop specific calibration subsets spectrally similar to each query sample whose properties are to be predicted and build a calibration model on the selected relevant samples for the query sample.

The key issue in local learning is how the similarity criterion should be constructed. In general, the most commonly used similarity checks in NIR spectroscopy are the Euclidean distance (ED) [15], the Mahalanobis distance (MD) [16], and spectral angle mapper (SAM) [17] distance on the spectral space or principal component space [18]. Moreover, the computation of the principal components-Mahalanobis (PC-M) [19] distance has become the standard procedure for NIR distance measurements. However, the samples are usually multivariate and influenced by several compositional attributes, which are expressed as highly overlapped and nonspecific NIR absorption or reflectance [20]. For this reason, the samples that are very close in *X* space are frequently not close (or similar) in terms of *Y* space. Therefore, traditional similarity criterion uses only *X* information to select relevant samples, which may result in a waste of *Y* information and inaccurate sample selection [21]. In order to overcome such problem, some supervised or semisupervised methods have been developed, which take account of information of both *X* and *Y*. Recently, another reliable similarity estimation called supervised locality preserving projection (SLPP) [22] has been successfully used in local approach to enhance the similarity measurement accuracy [21, 23].

As a simple linear approximation technique, local method could address the nonlinearity through the locally linear models. Therefore, the selection step of local method is aimed at finding some samples whose *X* and *Y* meet linear relationship. Unfortunately, similarity criterion mentioned above can only represent the closeness but not the true linear relationship between samples in spectra and property spaces.

The objective of the work described in this paper is to develop a high-performance local method for modelling NIR spectral data. The local errors regression utilizing SLPP and similar errors strategy in finding the optimized calibration subset for each query sample is described. The main contribution of this paper is to take into account the prediction errors from global method during the selection step of local method. This step ensures that the relationships between *X* and *Y* are highly linear in both calibration subset and query sample. After such a selection, PLS without cross-validation is adopted for local modelling as it can accelerate the prediction speed and reduce the computational complexity. With pharmaceutical tablets datasets of NIR spectra, the effectiveness and accuracy of the proposed method are investigated and compared.

## 2. Methodology

The local errors regression consisted of two steps: the first step is the selection of relevant samples and the second step is to build a calibration model for each query sample. In this section, the details of the proposed method and other algorithms for comparison are described.

### 2.1. Calibration Subset Selection

The main goal of this step is to discover which samples in calibration set “resemble” the query samples to be predicted. In the proposed method, the search process of calibration subset is carried out by using similarity of prediction errors which indicates how linear the spectra and property are to the samples for prediction. In this context, the parameter of error ranges need to be determined to ensure that there are sufficient selected samples to obtain reliable models. It should be noted that there might not be enough samples that are linear with the query sample to give an acceptable prediction. In this case, the definition of similarity, considering that information of both *X* and *Y* has been adopted to construct calibration subset. Although the proposed similarity criterion should be better than traditional method, it cannot be used directly to select the relevant samples for query samples as query samples only contain the spectra information. In this paper, an SLPP technique has been employed to find the nearest sample, whose properties are approximate and interchangeable with query sample.

Since the spectra *X* and property *Y* have different dimensions, it is not appropriate to find the nearest sample by the linear combination of EDs in spectral space and property space. Thus, the SLPP is used in this study, which not only selects most similar sample considering both spectra and property information but also reduces the computational load.

The rationale behind this approach is based on the assumption that the samples that are most close in the property space are very similar in terms of spectra space. Given a set of *N*-dimensional calibration spectral data *X*_*C*_ = [*x*_1_, *x*_2_,…, *x*_*n*_] in *R* ^*N*^ with corresponding property set *Y*_*C*_ = [*y*_1_, *y*_2_,…, *y*_*n*_], *N*-dimensional prediction spectral data *X*_*P*_ = [*x*_1_, *x*_2_,…, *x*_*m*_] ∈ *R* ^*N*^ with corresponding property set ${\hat{Y}}_{P}=[{\hat{y}}_{1},{\hat{y}}_{2},\ldots ,{\hat{y}}_{m}]$. Here *X*_*C*_ is *n* × *N* matrix containing *N* spectral responses of *n* samples; *X*_*P*_ is *m* × *N* prediction matrix containing *N* spectral data response of *m* samples; *Y*_*C*_ is *n* × *l* matrix; and *m* × *l* matrix ${\hat{Y}}_{P}$ is the predicted properties of prediction set with global PLS regression, *l* being the number of components. Let spectra set *X*_(*n*+*m*)×*N*_ = {*X*_*C*_ ∪ *X*_*P*_}. The algorithmic procedure for the calibration subset selection is stated as follows:

(1) Constructing a neighborhood graph as follows:
 
*K Nearest Neighbors*. The *i*th and the *j*th samples are connected by an edge if the *i*th sample is among *K* nearest neighbors of the *j*th sample or the *j*th sample is among *K* nearest neighbors of the *i*th sample. Here the distance between the samples is calculated in the property space.
(2) * Computing the Weights*. *W* is a sparse symmetric (*n* + *m*)×(*n* + *m*) matrix with *W*_*ij*_ having the weight of the edge joining the *i*th and the *j*th samples, and *W*_*ij*_ = 0 if there is no such edge, and *W*_*ij*_ = 1 if and only if the *i*th and the *j*th samples are connected by an edge.
(3) * Finding the Basis Vectors of the Subspace*. This step aims at finding a transformation matrix *A*_*N*×*d*_ to project spectra set *X* to a low-dimensional set *Z*_(*n*+*m*)×*d*_ = [*z*_1_, *z*_2_,…, *z*_*n*+*m*_] in *R* ^*d*^ (*d* ≪ *N*). Suppose that there exists a linear transformation *Z* = *Xa* ^*T*^, where *a* is the basis vector. The basis vector *a* is computed by solving the following minimization problem:
  (1) Min aXTWXaTSubject  to aXTXaT=1.
 It is observed that the minimization problem can be calculated by solving the following generalized eigenvalue problem:
  (2) $$\begin{array}{c} X^{T}LXa^{T}=\lambda X^{T}DXa^{T}, \end{array}$$
 where *D* is a diagonal matrix whose entries are column sums of *W* and *D*_*ii*_ = ∑_*j*_ *W*_*ji*_; *L* = *D* − *W* is the Laplacian matrix. Let the column vectors *a*_1_^*T*^,…, *a*_*d*_^*T*^ be the solutions to (2) which are ordered according to their eigenvalues, *λ*_1_ < ⋯<*λ*_*d*_. Thus, transformation matrix and low-dimensional matrix can be calculated as follows: *A* = [*a*_1_^*T*^,…, *a*_*d*_^*T*^], *Z*_*C*_ = *X*_*C*_ *A*, and *Z*_*V*_ = *X*_*V*_ *A*.

Finally, for each query sample, the most similar sample will be selected according to the ED in the low-dimensional space *Z*_*C*_.

### 2.2. Partial Least Squares Regression

The PLS regression has been extensively employed to obtain a quantitative model for prediction of analytes based on spectral data. In this paper, the PLS is used in the procedure of local selection and the establishment of regression model. In addition, the critical step is the determination of the number of factors for achieving the best prediction. Generally, the optimum PLS factors can be determined by minimizing the prediction error of cross-validation groups. However, the cross-validation is time-consuming and does not consider the information from the query sample. For these reasons it would not be a desirable choice to local method. In this paper, the selection of calibration subset is based on the prediction error with global PLS, which are expressed as highly linear between samples in subset. Consequently, the PLS factor would have little effect on the performance of calibration models. Here the PLS factor fixed at a constant value by minimizing the root mean squared error of prediction (RMSEP) of validation dataset and the experimental verification is presented in Section 4.1.

The performance of the final calibration models was evaluated in terms of the RMSEP, Residual Predictive Deviation (RPD), and correlation coefficient (*R* ^2^) in prediction set. It should be noted that RPD is the ratio between the standard deviation of the reference data and RMSEP of prediction.

### 2.3. Other Algorithms

In order to compare the predictive performance of our local errors method with other local approaches, the following similarity criterions were used: ED, angle distance, PC-M distance, and supervised or semisupervised methods. A brief description of these criterions is given as follows.

#### 2.3.1. Euclidean Distance

The ED between query sample *x*_*q*_ and calibration sample *x*_*i*_ is given by

(3) $$\begin{array}{c} d_{E}\left(\;x_{q},x_{i}\;\right)=\sqrt{{\left(x_{q}-x_{i}\right)}^{T}\left(x_{q}-x_{i}\right)}, \end{array}$$

where *T* is the transpose operation, *x*_*q*_ ∈ *X*_*P*_, and *x*_*i*_ ∈ *X*_*C*_.

#### 2.3.2. Angle Distance

In this method, the Cosine is used to evaluate the similarity between query data *x*_*q*_ and *x*_*i*_ in the spectral space, and the angle distance is defined as

(4) $$\begin{array}{c} d_{angle}\left(\;x_{q},x_{i}\;\right)=1-\frac{x_{q}\cdot x_{i}^{\prime }}{\sqrt{\left(x_{q}\cdot x_{q}^{\prime }\right)\left(x_{i}\cdot x_{i}^{\prime }\right)}}. \end{array}$$

#### 2.3.3. Mahalanobis Distance in the Principal Component

The PC-M distance is obtained through computing the Mahalanobis distance (MD) between samples in spectral principal component space. In this case, the appropriate number of principal components is determined by minimizing the root mean square of the compositional differences between property *Y* and predicted value $\hat{Y}$ of samples in calibration set. The Mahalanobis distance between query sample *x*_*q*_ and calibration sample *x*_*i*_ is defined as

(5) $$\begin{array}{c} d_{M}\left(\;x_{q},x_{i}\;\right)=\sqrt{{\left(x_{q}^{PC}-x_{i}^{PC}\right)}^{T}V^{-1}\left(x_{q}^{PC}-x_{i}^{PC}\right)}, \end{array}$$

where *V* is the covariance matrix in spectral principal component space of calibration set and *x*_*q*_^PC^ and *x*_*i*_^PC^ are spectra of query sample and calibration sample in spectral principal component space. For details, see [20].

#### 2.3.4. Supervised or Semisupervised Methods

*(1) Euclidean Distance Based on Both Spectra and Property*. In this method, both spectra *X* and property *Y* are used to evaluate the similarity between query data *x*_*q*_ and *x*_*i*_ in the calibration dataset, and the similarity value *d*_*XY*_ is defined:

(6) $$\begin{array}{c} d_{XY}\left(\;x_{q},x_{i}\;\right)=d_{E}\left(\;x_{q},x_{i}\;\right)\cdot \gamma +d_{E}\left(\;{\hat{y}}_{q},y_{i}\;\right)\cdot \left(\;1-\gamma \;\right), \end{array}$$

where *γ* is a weight parameter to balance the importance of spectra *X* and property *Y*, 0 ≤ *γ* ≤ 1, and *d*_*XY*_ is the Euclidean distance between *x*_*q*_ and *x*_*i*_. 

*(2) Euclidean Distance in the Low-Dimensional Space*. Here, the similarity measurement considers both *X* and *Y* information and utilizes SLPP technique to select relevant samples. For details, see [21].

In summary, the detailed implementation of local errors regression is described as follows.

**Step 1 .**

Compute the predicted properties of prediction set ${\hat{y}}_{P}$ and predicted properties of calibration set ${\hat{y}}_{C}$ with global PLS regression.

**Step 2 .**

For each query sample, select the most relevant sample from calibration set with SLPP.

**Step 3 .**

Predefine parameters such as PLS factors and error ranges by minimizing the RMSEP of validation dataset. Here PLS factors are set to 2, 3, 4, or 5 and error range is specified as 1.5. A much detailed description will be given in Section 4.1.

**Step 4 .**

For each query sample *y*_*i*_^*q*^, calibration subset is selected based on predicted error similarity criteria, and the *j*th sample in calibration set is selected if $\left|\left({\hat{y}}_{j}^{c}-y_{j}^{c})-({\hat{y}}_{i}^{q}-y_{i}^{q}\right)\right|<error\;\;range$, where *y*_*j*_^*c*^ is the property of the *j*th sample in calibration set.

**Step 5 .**

Build PLS calibration model on the selected relevant samples and predict each query sample.

It should be noted that the number of nearest neighbors *K* and dimension of transformation matrix *d* had little effect on the selection of the most similar sample for each query sample. Therefore, in Step 2, parameters *K* and *d* are set as 50 and 20, respectively. Such a selection is based on the authors' experience without further discussion.

## 3. Materials and Experiment

**Figure 1:**
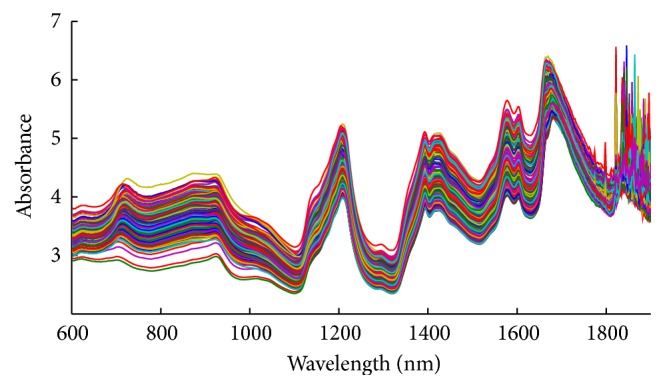
NIR original spectra for pharmaceutical tablets.

The dataset was obtained from the web of http://software.eigenvector.com/Data/tablets/index.html. The spectra were recorded by Instrument I of Foss NIR Systems 6500 Spectrometer. It contains 655 transmittance NIR spectra of pharmaceutical tablets, measured between 600 and 1898 nm with a 2 nm sampling step. The initial dataset was split into a calibration set of 460 samples and prediction set of 155 samples and validation set of 40 samples.  Table 1 showed the statistical results of pharmaceutical tablets. All sets have similar averages and standard deviations which indicate that all the datasets can be used to represent the main variability of pharmaceutical tablets. A microcomputer (Lenovo) with an Intel Core 2 processor was used for all the calculations. All the algorithms were implemented using MATLAB 2012b. The raw NIR spectra of pharmaceutical tablets were presented in Figure 1.

## 4. Results and Discussion

### 4.1. Parameter Setting

**Figure 2:**
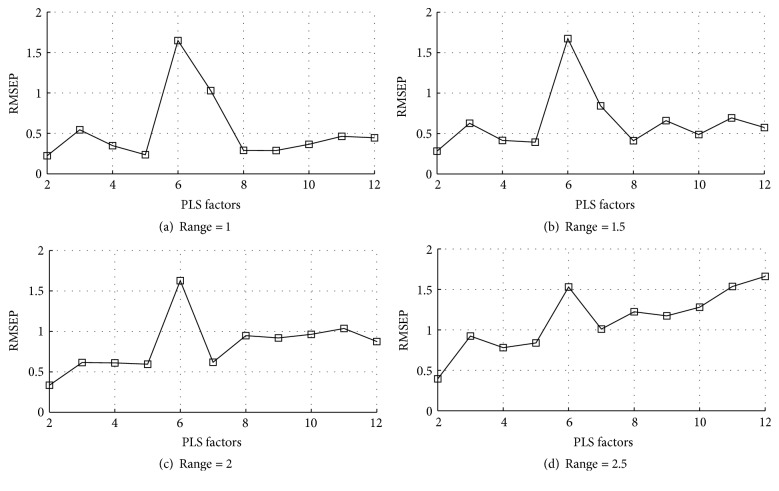
Variation of RMSEP with the PLS factors for validation set. RMSEP = root mean squared error of prediction. PLS = partial least squares.

The determination of the PLS factors and error ranges is an essential step in this study, which decides the accuracy of the model. Here these parameters were determined with the validation set so that the RMSEP was minimized. Therefore, the variation of the RMSEP of the validation set with PLS factors and error ranges is investigated.  Figure 2 shows the RMSEP obtained with PLS factors from 2 to 12 and a step of 1. For each query sample, the calibration subset is selected based on error ranges = 1, 1.5, 2, and 2.5, respectively. Different from the above narrative in Section 2.1, for each query sample, the most relevant sample is used instead of the real sample whose reference property is available in validation set. Moreover, when there are no enough samples with close similarity to the query sample, we assume that the predicted value is equal to the real reference property. The purpose of this assumption is to reflect the accurate relationship between the prediction performance and PLS factors with a fixed range.

**Figure 3:**
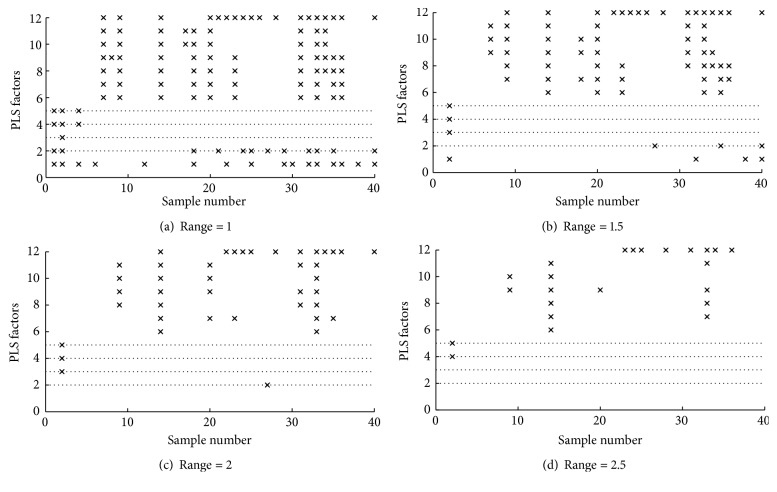
The probability of insufficient selected samples with the PLS factors and error ranges for the validation set. × = insufficient selected samples for building calibration model. PLS = partial least squares.

As shown in Figure 2, the smaller error range is employed, the smaller RMSEP can be achieved. Furthermore, when the PLS factors are 2 to 5, the RMSEPs change gradually with a little fluctuation. Other big PLS factors (i.e., greater than 7) are usually not necessary, and they do not lead to essential improvement of the final result. The probability of insufficient selected samples with the PLS factors and error ranges for the validation set is shown in Figure 3. It can be seen that the probability of insufficient selected samples for each query sample decreases along with error range, especially when the PLS factor is set as 2, 3, 4, and 5. In this study, considering both minimized RMSEP and the adequate size of the calibration subset, the error range is set as 1.5 and the PLS factors is set as 2, 3, 4, and 5, respectively. This means that the PLS factor will be set as 3, 4, and 5 in turn if there is no enough samples close to the query sample with PLS factor = 2.

### 4.2. Size of Calibration Subset

The final results of the local errors strategy for each query sample in prediction set are summarized in Table 2. It can be seen that each number of selected samples in calibration subset is smaller than 210, which is less than the half size of calibration set. Moreover, 78.7% sizes of calibration subsets are between 13 and 50. That means that the computational complexity of the prediction model can be reduced a lot compared with global method. It is noted that here the minimum size of the subset is set to 13 so as to avoid insufficient selected samples for building calibration model. When the number of selected samples is not reaching 13, the calibration subset has been made up of 50 samples which are the spectral similarity to query sample.

### 4.3. Comparison

**Figure 4:**
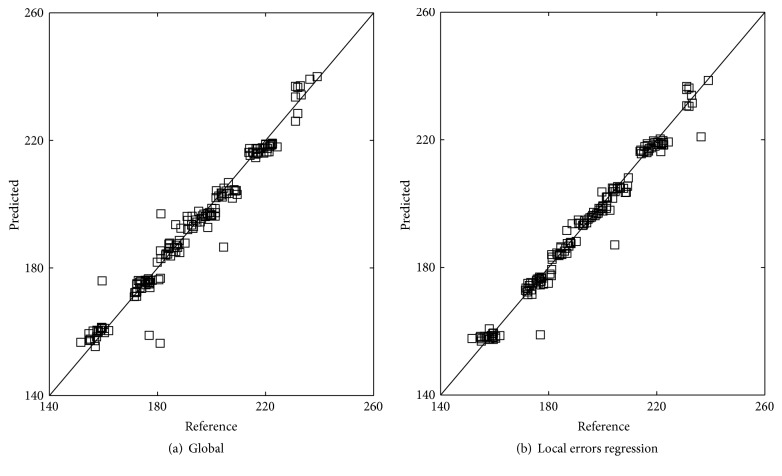
Predicted versus reference values of prediction set for the local errors regression and global method.

Figure 4 is the scatter plot that shows a correlation between reference values and NIR prediction values in the prediction set using global PLS method and proposed method. According to the scatter plots, the predicted values associated with local errors regression best match the reference ones. It can thus be concluded that the proposed method maintains good precision compared with global PLS method. Furthermore, the method proposed here was compared with other local methods.

The performance of local errors regression, global PLS, and other local methods with different similarity criterions was listed in Table 3. In each local-PLS model, two parameters have to be optimized: size of calibration subset and PLS factors. In this study, the number of calibration subset samples included was evaluated at 30, 50, 100, 150, 200, and 250 samples and checked so as to find which value for this parameter is better. The optimum number of PLS factors was identified by minimizing the prediction error of cross-validation groups. Best prediction results from local-PLS approaches with different similarity criterions are listed in the table. On the contrary, the PLS factors used in the proposed method, as discussed in the previous section, was fixed at either 2 or 3 or 4 or 5.

In terms of RMSEP, with the same spectra data the prediction performance of the local methods is better than that of global method, showing lower RMSEP values. Moreover, the RMSEP obtained with the proposed method is the lowest compared to the best values obtained with other local methods. Furthermore, Table 3 also shows that the difference of *R* ^2^ with different methods is not significant and all the values of *R* ^2^ are approximated to 1. The RPD is proportional to the RMSEP and with a similar variation tendency. Another important issue is the prediction speed. In this context all the local methods were slower than global method, showing higher time consumption. As shown in Table 3, global method requires around 4.4 s to calculate all the samples in prediction set and local methods are much more time-consuming, from 53.5 s to 233.9 s depending on subset size. The reason is that the local methods will select different calibration subset and perform new calibration models specifically to different query samples. Thus, in the proposed method, instead of cross-validation, the fixed PLS factor strategy is adopted, which resulted in a noticeable improvement of calculation speed, showing a lower time consumption about 9.5 s.

## 5. Conclusion

In this paper, a new local method for nonlinear spectra is proposed. The main contributions include a new similarity measurement utilizing ED of predicted errors, a selection method extended from SLPP aiming to search the most relevant sample for each query sample and fixed PLS factors prediction model without cross-validation. Also, a comparison has been carried out in this study, to compare the predictive performance of the proposed method, global PLS, and other local strategies with different similarity criterions such as ED, Cosine, PC-M, *X* + *Y* + ED, and *X* + *Y* + SLPP by using the pharmaceutical tablets dataset. The prediction results have demonstrated that the proposed method could bring higher calibration performances and lower time consumption.

## Figures and Tables

**Table 1:** Descriptive statistics for the calibration set, validation set, and prediction set.

Sample sets	Number	Range	Mean	Standard deviations
Calibration	460	154.3~237.7	188.4	15.8
Validation	40	168.2~219.5	194.8	12.4
Prediction	155	151.6~239.1	192.9	22.0

**Table 2:** Size of calibration subset for each query sample with local errors strategy in prediction set.

Number of samples in calibration subset	Number of query samples	Percentage
[13,50]	122	78.7%
[51,100]	7	4.5%
[101,150]	9	5.8%
[151,200]	11	7.1%
[201,210]	6	3.9%

**Table 3:** Performance comparisons among local errors regression, global method, other local methods with different similarity criterions.

Method	Similarity criterion	RMSEP	*R* ^2^	RPD	Size of subset	Parameters	Time consumption (s)
Global	—	4.30	0.96	5.11	460	—	4.4

Other local methods	ED	4.18	0.96	5.26	150	—	175.4
	Cosine	4.25	0.96	5.17	150	—	179.1
	PC-M	4.21	0.96	5.22	50	PC factors = 10	53.5
	*X* + *Y* + ED	4.24	0.96	5.18	100	*γ* = 0.8	123.1
	*X* + *Y* + SLPP	4.27	0.96	5.15	200	*γ* = 0.8, *d* = 20	233.9

Local errors regression	Errors + ED	3.21	0.98	6.85	13~205	*d* = 20	9.5

ED: Euclidean distance; PC-M: Principal components-Mahalanobis distance; *X* + *Y* + ED: Euclidean distance considering both spectra *X* and property *Y*; *X* + *Y* + SLPP: Euclidean distance in the low-dimensional space obtained with supervised locality preserving projection method; errors + ED: Euclidean distance between predicted errors; RMSEP: root mean squared error of prediction; *R* ^2^: correlation coefficient in prediction set; RPD: residual prediction deviation; PC factors: Principal component factors; symbol *γ*: a trade-off parameter to balance the importance of spectra *X* and property *Y*; *d*: dimension of transformation matrix; and s: second.
